# Outcomes of the Deployment of the Auto-Visual Acute Flaccid Paralysis Detection and Reporting (AVADAR) System for Strengthening Polio Surveillance in Africa From 2017 to 2018: Evaluation Study

**DOI:** 10.2196/18950

**Published:** 2020-12-02

**Authors:** Johnson Muluh Ticha, Godwin Ubong Akpan, Lara MF Paige, Kamel Senouci, Andrew Stein, Patrick Briand, Jude Tuma, Daniel Rasheed Oyaole, Reuben Ngofa, Sylvester Maleghemi, Kebba Touray, Abdullahi Ahmed Salihu, Mamadou Diallo, Sisay Gashu Tegegne, Isah Mohammed Bello, Umar Kabo Idris, Omosivie Maduka, Casimir Manengu, Faisal Shuaib, Michael Galway, Pascal Mkanda

**Affiliations:** 1 World Health Organization Regional Office for Africa Brazzaville Congo; 2 Bill and Melinda Gates Foundation Seattle, WA United States; 3 Novel-T Sarl Geneva Switzerland; 4 World Health Organization Geneva Switzerland; 5 World Health Organization Abuja Nigeria; 6 World Health Organization South Sudan Office Juba South Sudan; 7 University of Port Harcourt Port Harcourt Nigeria; 8 National Primary Health Care Delivery Agency (NPHCDA) Abuja Nigeria

**Keywords:** Auto-Visual Acute Flaccid Paralysis Detection and Reporting, surveillance, informants, acute flaccid paralysis, smartphones, polio

## Abstract

**Background:**

As we move toward a polio-free world, the challenge for the polio program is to create an unrelenting focus on smaller areas where the virus is still present, where children are being repeatedly missed, where immunity levels are low, and where surveillance is weak.

**Objective:**

This article aimed to describe a possible solution to address weak surveillance systems and document the outcomes of the deployment of the Auto-Visual Acute Flaccid Paralysis Detection and Reporting (AVADAR) project.

**Methods:**

This intervention was implemented in 99 targeted high-risk districts with concerns for silent polio circulation from eight countries in Africa between August 1, 2017, and July 31, 2018. A total of 6954 persons (5390 community informants and 1564 health workers) were trained and equipped with a smartphone on which the AVADAR app was configured to allow community informants to send alerts on suspected acute flaccid paralysis (AFP) and allow health worker to use electronic checklists for investigation of such alerts. The AVADAR and Open Data Kit ONA servers were at the center of the entire process. A dashboard system and coordination teams for monitoring and supervision were put in place at all levels.

**Results:**

Overall, 96.44% (24,142/25,032) of potential AFP case alerts were investigated by surveillance personnel, yielding 1414 true AFP cases. This number (n=1414) reported through AVADAR was higher than the 238 AFP cases expected during the study period in the AVADAR districts and the 491 true AFP cases reported by the traditional surveillance system. A total of 203 out of the 1414 true AFP cases reported were from special population settings, such as refugee camps and insecure areas. There was an improvement in reporting in silent health areas in all the countries using the AVADAR system. Finally, there were 23,473 reports for other diseases, such as measles, diarrhea, and cerebrospinal meningitis, using the AVADAR platform.

**Conclusions:**

This article demonstrates the added value of AVADAR to rapidly improve surveillance sensitivity. AVADAR is capable of supporting countries to improve surveillance sensitivity within a short interval before and beyond polio-free certification.

## Introduction

Cases of paralysis caused by poliovirus have decreased by 99% since the World Health Assembly’s resolution to eradicate polio [1]. The World Health Organization (WHO) recommends that a sensitive acute flaccid paralysis (AFP) surveillance system network should detect at least one case of nonpolio AFP annually per 100,000 children under 15 years of age. In addition, performance indicators for AFP surveillance require that reporting be timely and complete, and represent the geography of the country. [2]. All cases of AFP should be investigated and two stool samples should be collected from each AFP case at least 24 hours apart for viral isolation in a WHO-accredited laboratory [2]. The quality of AFP surveillance is therefore critical in countries moving toward the final phases of polio eradication [3]. Some countries in the African region have challenges with surveillance performance especially in security-challenged areas. For increasing AFP detection rates, innovations can be used to address challenges to the surveillance system and improve reporting in areas where key indicators of AFP surveillance are not being met, where surveillance is not being performed systematically, and where access to communities is a challenge. Studies have shown that the use of mobile technology for health in developing countries is an innovative and cost-effective approach to reach populations in low-resource settings [4]. Mobile phones are increasingly accessible worldwide [5]. In sub-Saharan Africa, the penetration of cell phones was estimated to be 63% in 2013 and projected to be more than 70% by 2015 [5]. Although mobile phone–based surveillance has the potential to provide real-time validated data for disease clustering and prompt response and investigation, little evidence is available on the current practice in sub-Saharan Africa [6]. In 2016, Shuiab et al demonstrated the use of an SMS text messaging–based technology app on smartphones called Auto-Visual AFP Detection and Reporting (AVADAR) that was given to health workers and community informants to improve the reporting of AFP in Nigeria [2]. Improvement in AFP reporting was observed in the pilot districts of Kuje and Oyun, demonstrating the added value of utilizing the AVADAR tool. With these initial positive results, AVADAR was implemented by the WHO Regional Office for Africa and used in 99 districts in Cameroon, Chad, the Democratic Republic of Congo, Liberia, Sierra Leone, South Sudan, and Niger, with technical support from the partner agencies Novel-T and eHealth Africa and funding provided by the Bill and Melinda Gates Foundation. The aim of this study was to determine the outcomes of the deployment of AVADAR for enhanced surveillance and reporting in these districts.

## Methods

### Selection of Countries

Eight countries were enrolled into the AVADAR system. The inclusion criteria were based on polio risk analysis performed from 2016 to 2018, which prioritized the following criteria for targeted surveillance interventions: endemicity for polio (Nigeria), countries with districts in the Lake Chad basin area (Cameroon, Chad, and Niger), history of recent Ebola virus disease (Sierra Leone and Liberia), and areas with insecurity and hard to reach populations (South Sudan and the Democratic Republic of Congo). Selection of countries was carried out by the WHO Regional Office in Africa in collaboration with the country governments.

### Selection of Districts

In each of the selected countries, a limited number of the highest risk districts for polio transmission were selected. The inclusion criteria used were as follows: weak surveillance system (as evidenced by a low nonpolio AFP rate; <2 for 100,000 children aged less than 15 years), existence of a telecommunications network in the community, presence of literate community members, presence of displaced populations or security challenges, and acceptance of the host governments for the use of technology in health. Selection was carried out by the health district authorities under supervision of the provincial and national health authorities in each country through final approval by the WHO Regional Office. An average of three to four districts were engaged per country. A total of 99 districts were included in the study

### Selection of Community Informants

An average of 130 to 150 informants were selected per health district. Informants were selected by district-level health authorities in collaboration with local community leaders. Informants were listed by the health areas where they lived, and efforts were made to ensure that informants were evenly spread across all the health areas. Informants were expected to be able to read and write at a basic school level, have previous field experience in immunization activities including polio eradication, be able to manipulate a mobile phone, and accept being part of the project. In Chad and Cameron, there were a high number of informants located in critical geographies for polio surveillance, who were unable to read or write. They were, however, able to recognize a suspected case of AFP. Thus, these “special informants” were recruited and included in the project. The final list was then uploaded on the AVADAR server to monitor reporting by the informants. All informants were geo-located through “zero reporting” and “home reporting” on their mobiles. Zero reporting was done weekly by all informants enrolled in the system for not finding any child with AFP [7] (Multimedia Appendix 1). Home reporting was done once after the informant was enrolled in the AVADAR system. The informant clicked an icon when at home, which automatically recorded the map position, and the information was stored on the AVADAR server. This served to display the geographic spread of informants in the health areas or districts selected for the AVADAR project, as well as the expected location (within a certain geographic range) where future reports would come from, although a community informant could report an alert beyond this geography.

### Selection of Health Workers

Health workers were selected in the same health areas and districts from where community informants were selected. One health worker was selected for every 10 community informants. Health workers (members of the existing surveillance system) were defined as those within a community with good experience in AFP surveillance or polio eradication activities and were motivated to conduct field investigations on suspected AFP cases (alerts). The health workers and informants were not paid. However, they were provided with air time (talk time) and data to send the weekly reports, and transportation costs to investigate alerts on suspected AFP cases were covered by the program.

### Training

A 2-day national training of trainers was organized in each country to inform, sensitize, and train central-level health authorities on the AVADAR system. This was followed by another 2-day training of health workers and informants on the AVADAR app with practical demonstrations, using the repetitive training methodology to ensure that both informants and health workers were fully aware of the alerting and investigation process and could function independently [8,9].

### Pilot Testing of the AVADAR System

The AVADAR system was piloted in two districts in Nigeria in 2016. The lessons learned from the pilot testing were used to improve checklists and selection of participants and to refine the training methodology. Various processes were thus adapted to country contexts as per health system requirements in terms of terminology and norms [10].

### AVADAR Servers and Investigation of Alerts

The actors in the AVADAR system (informants and health workers) were configured in the AVADAR server. On a weekly basis, the server auto-played the AFP case definition video. Each informant was expected to respond in order to confirm that he/she is still in the system. This was called a zero report. When an informant submitted a report, the report was assigned a unique number called report submission identification and an alert was sent to the AVADAR server. The AVADAR server then sent an automated SMS text message to health workers’ phones. On average, 10 health workers were configured to receive an SMS from the server for every AFP alert from an informant to ensure correct tracking and investigation of all alerts. Upon receipt of the SMS, the health worker and the informant could use the closed user group (CUG) telecommunications feature to further discuss the report. CUG is a network of SIM cards with unlimited access to make free phone calls to other SIM cards within the network. AVADAR informants and health workers investigating alerts were members of the CUG. A health worker traveled to investigate the case. Once the investigation was completed and the report was uploaded to the server, an SMS text message or email was sent from the server to preselected stakeholders to receive the findings of the investigation. As soon as the health worker confirmed that the alert investigated was a true AFP, further investigations were performed, leading to the collection of stool samples. In parallel, a second server (Open Data Kit [ODK]) received the same SMS text message. This ODK server has been designed to display some key predetermined outputs in the form of indicators.

### Data Collection and Use

A simple electronic questionnaire was developed and deployed on the smartphone of each informant. This enabled ease of use of the AVADAR app when a case was suspected by recording few key variables. These variables included the name of the suspected case (alert), location, and duration from onset to detection. Submission of the questionnaire to the server was by a simple click.

Similarly, two electronic checklists for health workers were built using ODK and downloaded onto their smartphones. One checklist collected data during investigation of alerts sent by informants. This is a critical stage in the AVADAR system because the alerts being investigated may turn out to be true AFPs. Once an investigation was completed, the checklist was uploaded to the sever. A trigger generated by the server was sent to a determined number of persons situated at the district, province, nation, or beyond. This trigger was a reminder that an alert has been investigated, and if the alert was a true AFP, follow-up was needed to ensure that a field investigation of the case was conducted timely. The second checklist for health workers on ODK was used to collect data during supervision of AVADAR activities.

### AVADAR Dashboard

The AVADAR dashboard reconciled all the soft documentation of alerts and investigations and evaluated the performance of the surveillance system in real time. This dashboard was an output of the reconciliation of the AVADAR server, which housed all informant reporting (zero and suspected AFP reporting) and the ODK server (the actual investigation by health workers). This dashboard made available in real time the status of all AFP alerts and investigations done via AVADAR from the country level to the ward/settlement level. It also assisted stakeholders to immediately assess where supervisory activities should be focused.

### Monitoring the Implementation of AVADAR

A major feature of AVADAR is that the platform is useful for monitoring the implementation of surveillance processes and activities in high-risk areas. In each AVADAR district, there was a coordination team consisting of an AVADAR coordinator from WHO, an eHealth technical coordinator, and a Ministry of Health focal person. This same structure existed at the ward level (health area) where the head of the health area coordinated all AVADAR activities. Monthly meetings were organized by the Ministry of Health with support from WHO and eHealth to monitor activity implementation and progress, and to solve problems related to smartphone functionality and chargers. National-, provincial-, and district-level officers conducted regular supportive supervisory visits for the various community informants to ensure that activities were being implemented as planned and to resolve challenges. The WHO Regional Office had regular teleconferences with countries, conducted field visits, and organized an annual review and planning meeting bringing together various stakeholders with objectives to foster program coordination and performance [11].

### Ethical Approval and Consent to Participate

Approval from an ethics committee and consent to participate were not required for analyses based solely on secondary data.

## Results

A total of 5390 informants were engaged in the AVADAR project in the eight countries from August 1, 2017, to July 31, 2018. Of the 5390 informants, 96.99% (n=5228) were community informants and 3.01% (n=162) were special informants (Table 1). The number of community informants who participated in the AVADAR system was dependent upon the number of health areas. Nigeria, which deployed AVADAR in 54 health districts, had 44.86% (n=2418) of the total informants, while South Sudan, which deployed AVADAR in three health districts, had 4.66% (n=251) of the total community informants. Similarly, a total of 1564 health workers were engaged in the project in the eight countries. The health workers engaged in Nigeria represented 55.88% (n=874) of the total health workers in the project, while those engaged in South Sudan represented 1.59% (n=25) of the health workers.

**Table 1:** Human capital and smartphones engaged for the Auto-Visual Acute Flaccid Paralysis Detection and Reporting (AVADAR) project in eight countries from August 1, 2017, to July 31, 2018.

Country	Community informants (N=5228), n (%)	Special informants (N=162), n (%)	Total informants (N=5390), n (%)	Health workers (N=1564), n (%)	Total (N=6954), n (%)
Nigeria	2418 (46.25%)	0 (0.00%)	2418 (44.86%)	874 (55.88%)	3292 (47.34%)
Chad	441 (8.44%)	104 (64.20%)	545 (10.11%)	77 (4.92%)	622 (8.94%)
Cameroon	552 (10.56%)	28 (17.28%)	580 (10.76%)	108 (6.91%)	688 (9.89%)
Niger	471(9.01%)	30 (18.52%)	501 (9.29%)	133 (8.50%)	634 (9.12%)
Liberia	288 (5.51%)	0 (0.00%)	288 (5.34%)	114 (7.28%)	402 (5.78%)
Sierra Leone	405 (7.75%)	0 (0.00%)	405 (7.51%)	98 (6.27%)	503 (7.23%)
Democratic Republic of Congo	402 (7.69%)	0 (0.00%)	402 (7.46%)	135 (8.63%)	537 (7.72%)
South Sudan	251 (4.80%)	0 (0.00%)	251 (4.66%)	25 (1.59%)	276 (3.97%)

A total of 162 special informants were engaged in three countries (Chad, Cameroon, and Niger) (Table 1). The special informants represented 2.33% (n=6954) of all informants engaged in the eight countries. The special informants in Chad constituted 16.7% (n=104) of all community informants engaged in the country (N=622). In Cameroon, the special informants represented 4.1% (n=28) of all informants in the country (N=688), and in Niger, the special informants represented 4.7% (n=30) of all informants engaged in the country (N=634). There was no difference in reporting alerts from both community informants and special informants.

Within the 12 months of the study, a total of 433,601 reports were expected to be sent by informants through weekly reports (also called zero reports) to indicate they were still in the network, and of these, 64.66% (280,376) were received (Table 2). Each informant reported once a week for the 52 weeks of the study period. Of the reports received, 65% (280,376) were received timely (within 48 h). The highest proportion of reports were received from South Sudan, where 92.58% (3932/4247) of informants actively responded to the weekly calls. This was followed by Niger, with 79.61% (18,498/23,236). The least active country was Nigeria, with just 57.31% (135,129/235,767) of reports received. With regard to alerts sent to the server on suspected AFP cases, a total of 25,032 were received, and 96.44% (24,142) of these were investigated.

Of these 24,142 alerts investigated, 5.86% (1414) yielded true AFP cases. This yield varied across countries, with the highest yield of 24.70% (589/2385) in Nigeria, followed by 16.84% (33/196) in South Sudan. Chad and Liberia both had the lowest yields of 0.98% (119/12,103) and 0.93% (15/1615), respectively.

**Table 2:** Performance of the Auto-Visual Acute Flaccid Paralysis Detection and Reporting (AVADAR) system compared with the traditional system of reporting acute flaccid paralysis cases from August 1, 2017, to July 31, 2018.

Country	Expected number of reports	Total number of reports received	Completeness (%) of reports received	Expected number of alerts investigated	Number of Investigations done	Investigation (%) of alerts received	Expected number of AVADAR^a^ AFP^b^ cases	Number of AVADAR AFP cases	Proportion (%) of AVADAR AFP cases	Expected annual number of AFP cases in AVADAR districts	Number of AFP cases reported by the traditional surveillance system
Nigeria	235,767	135,129	57.31	2562	2385	93.09	2385	589	24.70	45	279
Chad	35,558	22,833	64.21	12,321	12,103	98.23	12,103	119	0.98	23	48
Cameroon	37,266	26,880	72.13	2050	1946	94.93	1946	96	4.93	40	34
Niger	23,236	18,498	79.61	1005	986	98.11	986	103	10.45	24	85
Liberia	29,051	19,855	68.35	1888	1615	85.54	1615	15	0.93	20	0
Sierra Leone	38,009	32,667	85.95	416	393	94.47	393	27	6.87	16	15
Democratic Republic of Congo	30,467	20,582	67.55	4593	4518	98.37	4518	432	9.56	56	24
South Sudan	4247	3932	92.58	197	196	99.49	196	33	16.84	14	6
All countries	433,601	280,376	64.66	25,032	24,142	96.44	24,142	1414	5.86	238	491

^a^AVADAR: Auto-Visual Acute Flaccid Paralysis Detection and Reporting.

^b^AFP: acute flaccid paralysis.

With regard to the AFP yield, a total of 238 AFPs were expected from AVADAR districts within the study period. In terms of this number, 491 (206.1%) AFPs were reported by the traditional AFP system and 1414 (591.1%) AFPs were reported through the AVADAR system. It is important to note that an AFP was attributed to the system that first reported the case (either the AVADAR or traditional system) to avoid double counting of cases.

As shown in Table 3, from 2016 to 2018, there were 381 silent wards as the AVADAR intervention took off in different countries. After the stabilization of the intervention, in 2018, there was a 100% reduction in silent health areas in the Democratic Republic of Congo and Liberia. In absolute terms, there was improvement in reporting across all countries, with the lowest evidence-based improvement being in Chad (32%). This further supports AFP surveillance intensification that AVADAR elaborates, leading to reduction in silent areas [12].

**Table 3:** Impact of Auto-Visual Acute Flaccid Paralysis Detection and Reporting (AVADAR) in eight countries on the reduction in silent health areas in 2016-2018 compared with 2019 (aggregated comparison).

Country	Maximum number of silent wards in 2016-2018	Number of silent wards in 2019	Percentage reduction in silent wards in 2019
Cameroon	58	27	53%
Chad	63	43	32%
Democratic Republic of Congo	43	0	100%
Liberia	1	0	100%
Niger	19	10	47%
Sierra Leone	40	25	38%
South Sudan	12	5	58%
Nigeria	145	49	66%
All AVADAR^a^ countries	381	159	58%

^a^AVADAR: Auto-Visual Acute Flaccid Paralysis Detection and Reporting.

Considering the traditional AFP cases reported, the AVADAR cases, and the silent district reduction reported in the same period (Table 2 and Table 3), we have provided the *P* values for comparisons in Table 4. We ran paired sample *t* tests on information in Table 2 for comparing AVADAR cases and traditional AFP cases (Table 2 column 9 [numbers: 589, 119, 96, 103, 15, 27, 432, and 33] vs column 12 [numbers: 279, 48, 34, 85, 0, 15, 24, and 6]), and obtained *P*=.07 (two-tailed) and *P*=.04 (one-tailed), using Microsoft Excel 2016 (Microsoft Corporation).

We also ran paired sample *t* tests on information in Table 3 for the silent ward reduction (Table 3 column 2 [numbers: 58, 63, 43, 1, 19, 40, 12, 145, and 381] vs column 3 [numbers: 27, 43, 0, 0, 10, 25, 5, 49, and 159]) and obtained *P*=.07 (two-tailed) and *P*=.04 (one-tailed), using Microsoft Excel 2016.

**Table 4:** Comparison of mean, standard deviation, and *P* value of the Auto-Visual Acute Flaccid Paralysis Detection and Reporting (AVADAR) system and traditional surveillance system regarding acute flaccid paralysis reporting and silent wards (2017-2018).

Variable	Number of AVADAR^a^ AFP^b^ cases	Number of AFP cases reported by the traditional surveillance system	Maximum number of silent wards in 2016-2018	Number of silent wards in 2019
Mean	314.2222222	109.1111111	84.66666667	35.33333333
Variance	210043.6944	27912.11111	14119.75	2471.75
Observations	9	9	9	9
Pearson correlation	0.944806625	N/A^c^	0.979342284	N/A
Hypothesized mean difference	0	N/A	0	N/A
*df*	8	N/A	8	N/A
*t* stat	2.014828247	N/A	2.088810505	N/A
*P* (T<=t) one-tailed	0.039343527	N/A	0.035073851	N/A
*t* critical one-tailed	1.859548038	N/A	1.859548038	N/A
*P* (T<=t) two-tailed	0.078687054	N/A	0.070147702	N/A
*t* critical two-tailed	2.306004135	N/A	2.306004135	N/A

^a^AVADAR: Auto-Visual Acute Flaccid Paralysis Detection and Reporting.

^b^AFP: acute flaccid paralysis.

^c^N/A: not applicable.

A total of 203 AFP cases were reported in special populations, of which 44.3% (n=90) were from internally displaced persons/refugees (Table 5). Approximately 7.8% (n=43) of the cases reported from internally displaced persons/refugees were from Nigeria owing to a high number of internally displaced person/refugee camps in AVADAR districts in Nigeria. A total of 72% (31/43) of cases reported from Nomads were from Chad. Chad has nomadic populations in all the six AVADAR districts. A total of 90% (63/70) of cases reported from areas of insecurity were reported from Nigeria that runs AVADAR in 54 local government areas with security challenges.

**Table 5:** Acute flaccid paralysis reported through Auto-Visual Acute Flaccid Paralysis Detection and Reporting (AVADAR) in special population settings (August 1, 2017, to July 31, 2018).

Country/special population	Number of AFP^a^ cases reported from IDPs^b^/refugees (N=90), n (%)	Number of AFP cases reported from Nomads (N=43), n (%)	Number of AFP cases reported from insecure areas (N=70), n (%)	Total (N=203), n (%)
Nigeria	43 (48%)	4 (9%)	63 (90%)	110 (54%)
Chad	0 (0%)	31 (72%)	0 (0%)	31 (15%)
Cameroon	16 (18%)	0 (0%)	5 (7%)	21 (10%)
Niger	31 (34%)	8 (19%)	0 (0%)	39 (19%)
South Sudan	0 (0%)	0 (0%)	2 (3%)	2 (1%)

^a^AFP: acute flaccid paralysis.

^b^IDP: internally displaced person.

Using the CUG network, informants were able to report other disease conditions apart from AFP. Nigeria reported the highest number of suspected measles cases (587/902, 65%) and suspected cerebrospinal meningitis cases (182/194, 93.8%). Similarly, the Democratic Republic of Congo reported 19,419 cases (86.8%, N=22,377) of acute watery disease (Table 6).

**Table 6:** Other disease conditions reported by Auto-Visual Acute Flaccid Paralysis Detection and Reporting (AVADAR) informants (August 1, 2017, to July 31, 2018).

Country	Number of suspected measles cases (N=902), n (%)	Number of acute watery disease cases (N=22,377), n (%)	Number of suspected cerebrospinal meningitis cases (N=194), n (%)	Total (N=23,473), n (%)
Nigeria	587 (65.1%)	1232 (5.5%)	182 (93.8%)	2001 (8.5%)
Chad	117 (13.0%)	133 (0.6%)	0 (0.0%)	250 (1.1%)
Liberia	60 (6.7%)	1550 (6.9%)	0 (0.0%)	1610 (6.9%)
Democratic Republic of Congo	133 (14.7%)	19,419 (86.8%)	12 (6.2%)	19,564 (83.3%)
South Sudan	5 (0.6%)	43 (0.2%)	0 (0.0%)	48 (0.2%)

## Discussion

### Principal Findings

We found a marked increase in the number of cases of AFP reported through the AVADAR system compared with those reported through the traditional system in the same districts within the same period. These findings are similar to those of Shuaib et al in the AVADAR pilot study in Kwara and Kuje LGAs in 2016 [2]. We also demonstrated that 14.36% (203/1414) of AFP cases were from areas with security challenges or from special populations. It is likely that without AVADAR, these cases would have been missed. In 2017, in a study on the use of mHealth in polio eradication and other immunization activities in developing countries, Kim et al concluded that growing access to technology and widespread mobile connectivity offer a tremendous opportunity for the immunization community to leverage these efforts to improve and sustain immunization services, particularly for populations currently not reached and at the highest risk of vaccine preventable diseases [13]. Other studies have also demonstrated the usefulness of several mHealth interventions for improved health care delivery at all levels of care and even under unfavorable security and economic conditions [14].

We also found statistical significance (*P*=.04) when comparing AVADAR AFP cases and traditional AFP cases for the period under review.

This study also demonstrated a marked reduction in silent health areas and districts in all the countries where AVADAR was deployed. This reduction in silent health areas was more significant in the Democratic Republic of Congo and Liberia. We also did a paired two samples test for means of silent wards before AVADAR and after AVADAR, which revealed reduction of silent districts based on the introduction of AVADAR at the district level. The output of the paired *T* sample analyses indicated that the mean for the silent districts before AVADAR/introduction of AVADAR was 84.67 and after AVADAR was 35.33. For our results, we used *P* (T<=t) two-tailed, which is the *P* value for the two-tailed form of the *t* test. Because our *P* value (.03) was less than the standard significance level of .05, we could reject the null hypothesis. Our sample data support the hypothesis that the before and after AVADAR silent district means are different.

The use of technology has been shown to improve health worker accountability and reporting. This is partly due to the removal of reporting barriers and the monitoring and peer review role of technology for improving data quality and reporting [15,16]. These qualities make AVADAR and other mHealth innovations useful for improving health outcomes [17].

The successful use of community informants and special informants indicates that AVADAR and other mHealth innovations when correctly deployed can be used even by nonhealth workers. The AVADAR program has a simplified case definition and has user-friendly forms and questionnaires suitable for nonhealth personnel. The departure from health worker–dependent surveillance to involving community informants has been shown to improve the quality and effectiveness of surveillance activities, especially in security-challenged locations [18].

We also found out that other disease conditions, such as measles, acute watery disease, and cerebrospinal meningitis, were reported through AVADAR. This is an indication that this community-based initiative (AVADAR) has the potential to report other disease conditions in a timely manner to trigger a timely response. Though AVADAR was originally designed for AFP surveillance, the platform could be used for other disease surveillance, demonstrating the use of the AVADAR platform beyond poliomyelitis eradication certification. This implies that the same infrastructure that supports AVADAR can be deployed to support other disease surveillance activities across countries. This makes the AVADAR system a robust and cost-effective solution [2].

The strength of this study is that it demonstrates the usefulness and impact of an mHealth solution like AVADAR at scale. This is unlike many other studies that only showed success with pilot studies or studies with small sample sizes and confined populations [6,19,20]. AVADAR is therefore proven to be a promising strategy for countries facing weak surveillance systems related to insecurity or those with hard-to-reach populations.

### Limitations

The use of AVADAR is limited by the weak telecommunication infrastructure in most countries, notably in rural and remote areas, which are the most suited for its application. Second, the system is relatively costly and thus heavily reliant on funding. Despite these limitations, AVADAR could be recommended in areas with surveillance gaps and challenges. However, government ownership and domestic funding are required to ensure sustainability.

### Conclusions

The marked increase in the case detection of AFP justifies the cost-intensive nature of the intervention. The system is relatively costly and thus heavily reliant on funding. Despite the high cost, AVADAR could be recommended in areas with surveillance gaps and challenges. However, government ownership and domestic funding are required to ensure sustainability.

AVADAR is recommended beyond the Global Polio Eradication Initiative program for broader public health initiatives and strengthening of health systems. The potential to keep on using the structure set up with minimal additional infrastructure cost also makes this intervention a worth-while solution for enhanced surveillance and control of communicable and noncommunicable diseases.

Within a 12-month period, AVADAR showed a positive impact by improving AFP surveillance performance to certification standards. Achieving certification standards remains a key requirement for all districts, and the African region achieved a wild polio–free status on August 25, 2020.

## Appendix

Multimedia Appendix 1 AVADAR video for stimulating and reminding Diseases Notification Officers to conduct active surveillance and submit weekly AFP or Zero reporting.

## Abbreviations

AFP: acute flaccid paralysis
AVADAR: Auto-Visual Acute Flaccid Paralysis Detection and Reporting
CUG: closed user group
ODK: Open Data Kit
WHO: World Health Organization
